# Phylogenetic confirmation of the genus *Robbea* (Nematoda: Desmodoridae, Stilbonematinae) with the description of three new species

**DOI:** 10.1080/14772000.2014.941038

**Published:** 2014-08-07

**Authors:** Jörg A. Ott, Harald R. Gruber-Vodicka, Nikolaus Leisch, Judith Zimmermann

**Affiliations:** ^a^ Department of Limnology and Biooceanography, University of Vienna, Althanstr. 14, A-1090Vienna, Austria; ^b^ Department of Symbiosis, Max Planck Institute for Marine Microbiology, Celsiusstr. 1, D-28359Bremen, Germany; ^c^ Department of Ecogenomics and System Biology, University of Vienna, Althanstr. 14, A-1090Vienna, Austria

**Keywords:** Belize Barrier Reef, Caribbean Sea, chemosynthetic symbiosis, marine nematodes, molecular phylogeny, SSU rRNA, ectosymbionts, systematics, taxonomy

## Abstract

The Stilbonematinae are a monophyletic group of marine nematodes that are characterized by a coat of thiotrophic bacterial symbionts. Among the ten known genera of the Stilbonematinae, the genus *Robbea* Gerlach 1956 had a problematic taxonomic history of synonymizations and indications of polyphyletic origin. Here we describe three new species of the genus, *R. hypermnestra* sp. nov., *R. ruetzleri* sp. nov. and *R. agricola* sp. nov., using conventional light microscopy, interference contrast microscopy and SEM. We provide 18S rRNA gene sequences of all three species, together with new sequences for the genera *Catanema* and *Leptonemella*. Both our morphological analyses as well as our phylogenetic reconstructions corroborate the genus *Robbea*. In our phylogenetic analysis the three species of the genus *Robbea* form a distinct clade in the Stilbonematinae radiation and are clearly separated from the clade of the genus *Catanema*, which has previously been synonymized with *Robbea*. Surprisingly, in *R. hypermnestra* sp. nov. all females are intersexes exhibiting male sexual characters. Our extended dataset of Stilbonematinae 18S rRNA genes for the first time allows the identification of the different genera, e.g. in a barcoding approach.

http://zoobank.org/urn:lsid:zoobank.org:pub:D37C3F5A-CF2B-40E6-8B09-3C72EEED60B0

## Introduction

Stilbonematinae are a marine subfamily within the nematode family Desmodoridae (order Desmodorida) and occur worldwide in sulphidic sediments (reviewed in Tchesunov, 2013). They are characterized by an ectosymbiosis with sulphur-oxidizing bacteria that covers their cuticle in a genus- and sometimes even species-characteristic manner (Polz *et al.*, 1992, 1994; Ott *et al.*, 2004). Although the family Desmodoridae is probably polyphyletic (van Megen *et al.*, 2009), molecular data (Kampfer *et al.*, 1998; van Megen *et al.*, 2009) suggest a monophyly of the subfamily Stilbonematinae. A distinct morphological synapomorphy of the subfamily is the complex glandular sense organ (GSO) described by Nebelsick *et al*. (1992) and Bauer-Nebelsick *et al*. (1995), that has so far only been found in members of the Stilbonematinae. This organ produces a special set of lectins (sugar-binding proteins) that seem to be involved in sustaining the specificity of the symbiosis (Bulgheresi *et al.*, 2006, 2011). Otherwise very few common features unite the group, such as the lack of a buccal armature and the weak development of the pharynx (Ott *et al*., 2004). Some characters appear to have developed independently more than once. For example, there is a tendency towards the development of an enlarged muscular portion of the anterior part of the pharynx (corpus), which is conspicuous in all species of the genera *Robbea* and *Catanema*, and has been described for the single species of *Parabostrichus* (Tchesunov *et al*., 2012), whereas in other genera (*Laxus*, *Leptonemella*, *Eubostrichus*) it is present only in a few species. Other examples are the reduction of the amphidial fovea or the presence of a stiff corpus gelatum protruding from the amphidial fovea (*Stilbonema*, *Catanema, Leptonemella* partim) (Tchesunov, 2013). This complicates the assessment of the relationships between the various genera described until now.

In addition, the type species of several genera are either inadequately described or have features which are the exception rather than the rule in the subsequently described members of the genus. Liberal synonymization has added further confusion.

The genus *Robbea*, introduced by Gerlach (1956), is characterized by the clear separation of a muscular anterior corpus from a mostly glandular isthmus and posterior bulbus which make up the remaining portion of the pharynx. In some species the males have peculiar papillae in the ventro-median line of the cervical and post-cervical region, which resemble suction cups and have a stout conical setae in its centre. These appear to be unique for the genus *Robbea* and have not been described from other stilbonematine genera.

The aim of this study was to reinvestigate morphological characters that are shared by all species of the genus *Robbea* but clearly distinguish those from other stilbonematine nematode genera. We describe three new species of *Robbea* from shallow subtidal sands around the island Carrie Bow Cay on the Belize Barrier Reef (Caribbean Sea). We combine morphological and molecular analyses to clarify the status of the genus and support our findings by providing additional phylogenetic information on previously under-sampled genera.

## Materials and methods

**Fig. 1.**
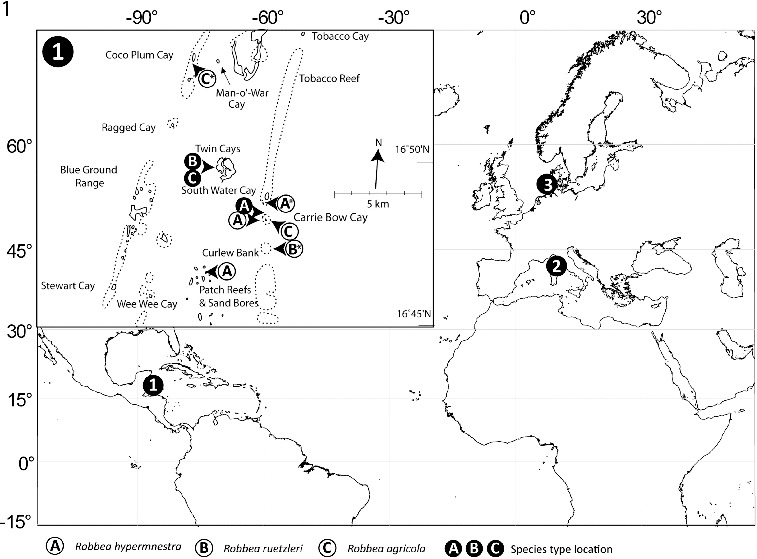
Map showing sampling locations for the nematodes used in this study. 1, Carrie Bow Cay, Belize; 2, Elba, Italy; and 3, Sylt, Germany. Insert shows detailed locations in the vicinity of Carrie Bow Cay where the three new *Robbea* species were found. Asterisks mark the sampling locations of the individuals that were used for sequencing.

Sediment samples containing *Robbea* species were collected in the vicinity of the island of Carrie Bow Cay, Belize from shallow subtidal sand patches and from turtle grass (*Thalassia testudinum*) beds using buckets or cores. Samples for sequencing were obtained at the following coordinates: *R. hypermnestra*: 16° 48′ 47″N, 88° 04′ 58″W; *R. agricola:* 16° 52′ 58″N, 88° 07′ 11″W; *R. ruetzleri:* 16° 47′ 19″N, 88° 04′ 48″W (Fig. 1). Subtidal sediment containing *Catanema* sp. was collected in the bay off Capo di Sant’ Andrea, Elba, Italy (42° 48′ 26″N, 10° 08′ 28″E) from sand patches surrounding sea grass beds of *Posidonia oceanica* in 4–8 m water depth (Fig. 1). *Leptonemella vicina* was collected off the island of Sylt, Germany (55° 00′ 54″N, 08° 26′ 16″E) with sediment cores from an intertidal sand flat (Fig. 1). Worms were extracted from the sand after anaesthesia with MgCl_2_, shaking and decanting or simply shaking and decanting through a 32 μm mesh sieve. The live animals were sorted using a dissecting microscope and immediately fixed in 4% formaldehyde (for taxonomic preparations) or 2.5% glutaraldehyde in 0.1 sodium cacodylate buffer and postfixed in 2% OsO_4_ (for SEM and semi-thin sections). For light microscopy the worms were placed into glycerol:water 1:9, slowly evaporated and finally mounted in pure glycerol; for SEM specimens were critical point-dried and coated with gold. Specimens for sequencing of the 18S rRNA gene were preserved in 70% ethanol or methanol.

Drawings were done using a *camera lucida* on a Reichert Diavar or a Leitz Ortholux. Nomarsky interference contrast photos were taken on a Reichert Polyvar; SEM on a JEOL JSM-35 CF and a Phillips XL20; semi-thin sections were cut on a Reichert Ultracut and photographed in a Reichert Polyvar.

### DNA extraction, PCR amplification and sequencing of the 18S rRNA gene

DNA for all sampled species was extracted and purified from single nematode specimens as described before (Schizas *et al.*, 1997). The partial 18S rRNA gene (∼1800 bp) was amplified by PCR with the general eukaryotic primers 1F (5′-GGTTGATYCTGCCAGT-3′) (modified from Winnepenninckx *et al.*, 1995) and 2023R (5′-GGTTCACCTACGRAAA-3′) (modified from Pradillon *et al.*, 2007) using the Phusion® DNA polymerase (Finnzymes, Finland). Cycling conditions were as follows: initial denaturation at 96 °C for 5 min, followed by 35 cycles of 96 °C for 1 min, 55 °C for 1.5 min, 72 °C for 2 min and final elongation at 72 °C for 10 min. The purified PCR products (PCR Purification Kit; Qiagen, Hilden, Germany) were sequenced bidirectionally with the PCR primers and an internal reverse primer (5′-CAGACAAATCGCTCC-3′) 1272 nucleotides downstream of the 1F primer. All sequencing reactions were performed with an ABI PRISM 3100 genetic analyser (Applied Biosystems, Foster City, CA, USA).

The generated 18S rRNA gene sequences and all sequences from the Stilbonematinae available in GenBank, as well as four Draconematinae sequences as outgroup were aligned using MAFFT Q-INS-I, which considers the predicted secondary structure of the RNA for the alignment (Katoh *et al.*, 2005). Alignments were manually inspected and 5′ and 3′ end-trimmed using Geneious software version 6 (Drummond *et al.*, 2011). The optimal substitution model was assessed using the Akaike information criterion as implemented in MEGA 5.3 (Tamura *et al.*, 2011) and the GTR+G+I model was chosen. Phylogenetic trees were reconstructed using maximum likelihood- (RAxML) (Stamatakis, 2006) and Bayesian inference-based (MrBayes) (Ronquist & Huelsenbeck, 2003) methods. The dataset was screened for chimeric sequences by calculating maximum likelihood-based trees on three partitions of the alignments (0–600 bp, 601–1200 bp and 1201–1799 bp). The Stilbonematinae sequences Y16915 (designated *Eubostrichus dianae*) and Y16921 (designated *Robbea hypermnestra*) had statistically supported positions in 2 (Y16915) and 3 (Y16921) different genus level clades across all three partitions and were excluded from the final analysis. MrBayes was run for four Mio generations using four chains. Convergence was evaluated by plotting the generations versus logL and the burn-in was set to 1 Mio generations. Node stability was evaluated using posterior probabilities (pp, Bayesian inference) and bootstrap support (200 RAxML rapid bootstrap runs, maximum likelihood) (Stamatakis *et al.*, 2008) with values above .80 considered significant.

### Nucleotide sequence accession numbers

The 18S rRNA sequences from this study were submitted to GenBank under accession numbers KJ414464 (*Robbea agricola*), KJ414465 (*Robbea ruetzleri*), KJ414466-7 (*Robbea hypermnestra*), KJ414468 (*Leptonemella vicina*) and KJ414469 (*Catanema* sp.).

## Results

Class Chromadorea Inglis, 1983

Subclass Chromadoria Pearse, 1942

Order Desmodorida De Coninck, 1965

Suborder Desmodorina De Coninck, 1965

Superfamily Desmodoroidea Filipjev, 1922

Family Desmodoridae Filipjev, 1922

Subfamily Stilbonematinae Chitwood, 1936

***Robbea*** Gerlach 1956

###  

#### Diagnosis

(Modified from Tchesunov, 2013): Stilbonematinae. Cuticle transversely striated, except for the head region and the tip of the tail. Cephalic capsule when present with a block-layer; cephalic setae as long or longer than the subcephalic setae, usually directed straight forward. Amphidial fovea well developed, spirally coiled or loop-shaped. Pharynx distinctly tripartite, corpus muscular and clearly set off from the narrow isthmus. Pharynx bulbus largely glandular. Gubernaculum variable in shape, with or without dorso-caudal apophysis. The males of *R. tenax* Gerlach 1963 and the three new species have cup-shaped ventral papillae in the post-pharyngeal region. These species share also a stout body with *a* not exceeding 100. The species *R. gerlachi* Boucher 1975 from which only a female has been described may also belong to this group. Symbiotic bacteria rod shaped, corn-kernel shaped or coccoid, usually covering the body as a monolayer.

***Robbea hypermnestra*** sp. nov.

http://zoobank.org/urn:lsid:zoobank.org:act:505DEA0B-614A-4086-A52F-62BA8A68F474

#### Synonymy

*Robbea* sp. in: Ott & Novak (1989), Schiemer *et al*. (1990), Ott *et al.* (1991), Polz *et al*. (1992), Bauer-Nebelsick *et al*. (1995), Urbancik *et al*. (1996a, 1996b), Polz *et al*. (2000); *Robbea* sp. 3 in: Bayer *et al*. (2009), Heindl *et al*. (2011); *Robbea hypermnestra* nomen nudum in: Kampfer *et al*. (1998), Polz *et al*. (2000).

#### Type material

Holotype (male), 4 paratypes (male), 4 paratypes (female/intersex), deposited at the US National Museum, Washington, D.C. (accession numbers USNM 1231551-1231559).

#### Measurements

See Table 1.

**Table 1.** Morphometric data for *Robbea hypermnestra* sp. nov. Ranges are given for the male and female paratypes. All measurements are in μm.

	Holotype	Paratypes (male) n = 4	Paratypes (female intersexes) n = 4
Length	3125	2765–3535	3020–3985
a	44.5	41.4–58.9	44.4–64.3
b	30.6	23.6–37.2	27.5–36.2
c	17.9	17.4–23.6	23.0–26.9
maximum width	70	55–65	65–82
pharynx length	102	92–100	90–117
tail length	174	140–162	120–150
nerve ring (% pharynx length)	50	48–54	46–58
corresponding body diameter (cbd)	45	45–52	48–55
bulbus length/width	25/32	20–28/29–30	25–38/32–45
bulbus cbd	48	47–50	48–55
V	n.a.	n.a.	41–45
vulva cbd	n.a.	n.a.	65–82
anal (cloacal) body diameter	68	55–65	50–60
tail length:anal diameter	2.6	2.3–2.9	2.3–2.5
spiculae length arc/chord	105/88	98–102/80–85	62–70/53–60
gubernaculum length/apophysis length	20/25	20–30/25–34	10–15/10–15
amphid width	15	16–20	9–12
cephalic setae number/length	4/38	4/32–40	4/30–38
subcephalic setae 1 number/length	8/37	8/30–38	8/28–32
subcephalic setae 2 number/length	8/30	8/28–32	8/28–32
Sucker-like papillae, number	17	16–18	n.a.
Position of first/last papilla	85/501	65–95/440–480	n.a.

n.a. = not applicable.

#### Additional material

Several specimens in the author's collection and those used for SEM.

The sequences of the 18S rRNA gene are available from GenBank and have the accession numbers KJ414466-7.

#### Type locality

Subtidal sand bar in 10–50 cm depth at the north end of Carrie Bow Cay, Belize in coarse, poorly sorted calcareous sand with rubble (Fig. 1).

#### Distribution

This robust species is extremely common in the above sand bar (compare Ott & Novak, 1989) and a similar sand bar at the south end of neighbouring South Water Cay. Other localities are open sand patches between *Thalassia testudinum* beds and patch reefs to the west and north of Carrie Bow Cay in up to 1.5 m depth and medium, poorly sorted sand at the base of ‘sand bores’ south of Carrie Bow Cay in 5–6 m depth (Fig. 1).

#### Etymology

Named after Hypermnestra, the only one of the 50 daughters of the king of Argos, Danaos, who did not kill her husband in the wedding night as requested by her father although she was armed with a dagger. This refers to the presence of a spiculum in females (intersexes).

### Description

**Figs. 2–10.**
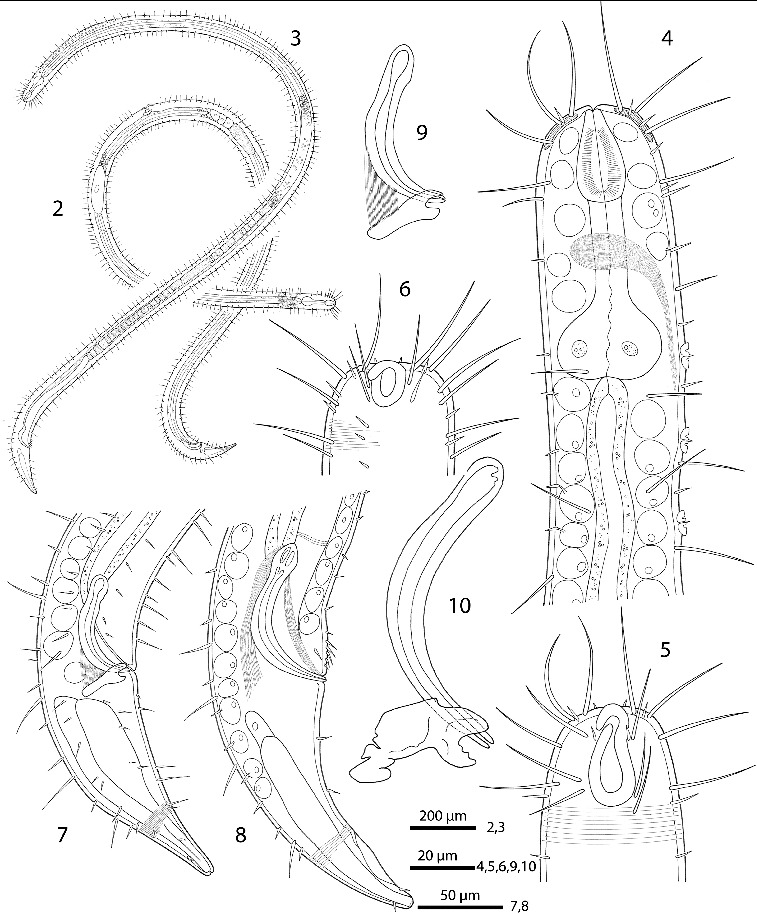
*Robbea hypermnestra* sp. nov. **2**. Female, whole; **3**. Male, whole; **4**. Male, anterior body region; **5**. Male, head, surface view; **6**. Female, head, surface view; **7**. Female, tail; **8**. Male, tail (gubernaculum not drawn); **9**. Female, spiculum; **10**. Male, spiculum.

Body cylindrical (Figs 2, 3), head diameter at level of cephalic setae 20–23 μm, diameter at level of posterior margin of amphidial fovea 40–54 μm in males, 33–42 μm in females, at end of pharynx 47–55 μm, maximum body diameter 55–82 μm, anal diameter 55–68 μm in males, 50–60 μm in females. Tail conical, 140–174 μm long in males and 120–150 μm in females (Figs 7, 8).

**Figs. 11–16.**
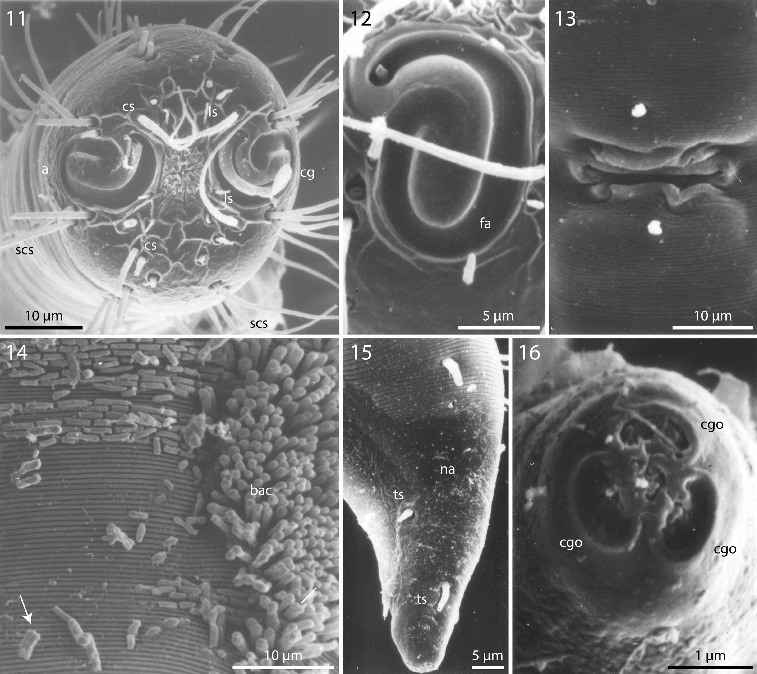
*Robbea hypermnestra* sp. nov. Female. **11**. Head, in face view; **12**. Amphidial fovea; **13**. Vulva; **14**. Annulation and bacterial coat in midbody region; **15**. Tip of tail, non-striated portion; **16**. Openings of the caudal glands. SEM.

**Figs. 17–26.**
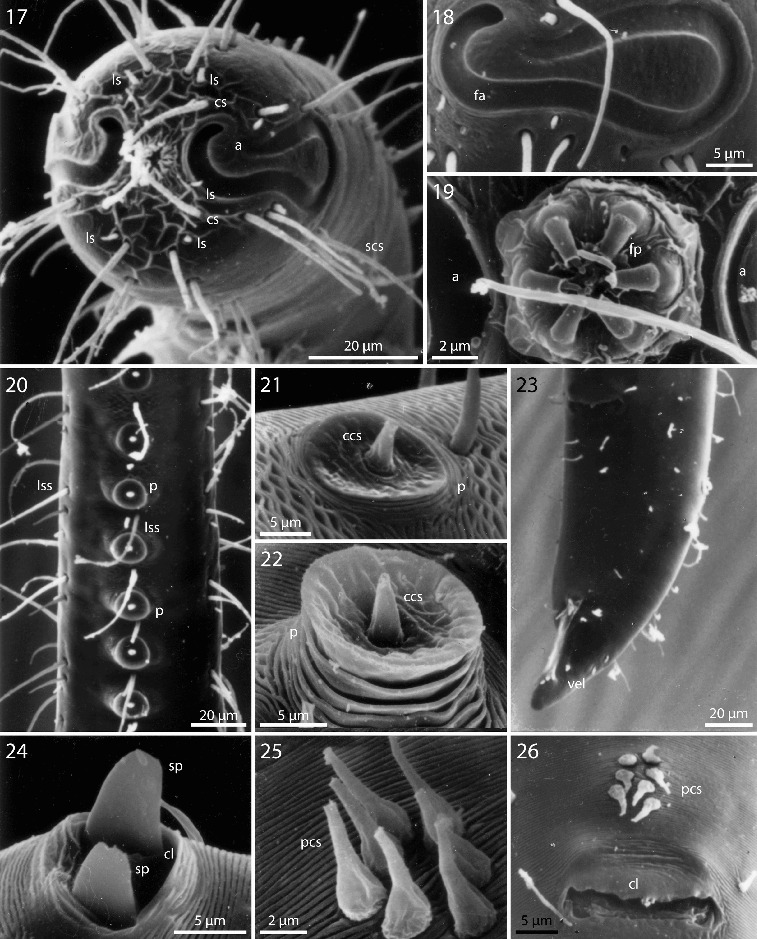
*Robbea hypermnestra* sp. nov. Male. **17**. Head, in face view; **18**. Amphidial fovea; **19**. Mouth opening with fingerlike papillae; **20**. Row of ventral sucker-shaped papillae; **21**. Papilla withdrawn; **22**. Papilla extended; **23**. Tail; **24**. Tips of spicule protruding from cloaca; **25**. Group of precloacal setae; **26**. Cloaca and precloacal setae. SEM.

Cuticle finely transversely striated except for the first 28–36 μm of the head (Figs 5–8, 14) and the last 35–50 μm of the tail (Fig. 15), annules 0.45 to 0.55 μm wide (18–22 annuli/10 μm); non-striated head cuticle reinforced (cephalic capsule). The anteriormost circle of head sensillae (inner labial sensillae) is represented by 6 finger-like papillae, 2 μm long in lateral, subventral and subdorsal position surrounding the mouth opening (Fig. 19). The second circle consists of 6 short outer labial sensillae, 3–6 μm long, on the margin of the membranous buccal field; 4 cephalic setae flanking the anterior margin of the amphidial fovea, 32–38 μm long; 3 circles of 8 subcephalic setae each, 28–38 μm long, on the non-striated part of the head region, the posteriormost at the level of the posterior margin of the amphidial fovea (Figs 11, 17); 8 rows of somatic setae along the whole length of the body, 20–35 μm long somatic setae alternating with only 5–12 μm long bristles (Fig. 4). In males, there is a distinct field of about 7–10 stout, conical setae, 5 μm long on the ventral side in front of the cloacal opening (Figs 8, 25, 26). Non-striated part of the tail in females with 2 pairs of stout setae, the first pair 7 μm long, shortly behind the end of the annulation; the second pair of 3–4 μm long, close to the tip of the tail (Fig. 15); in males with a small velum ventrally (Fig. 23). Three caudal glands with separate openings at the tail tip (Fig. 16). Males have a 380–450 μm long row of 16–19 (mean 17.2, n = 16) conspicuous cuticular papillae along the mid-ventral line beginning at a distance of 65–95 μm from the anterior end at the level of the posterior bulbus of the pharynx (Figs 4, 20). Papillae cup-shaped, with a diameter of 11–15 μm, on short annulated stalks, bearing a conical seta in its centre (Figs 21, 22). Amphidial foveas situated at the anterior end bordering the buccal field, showing a distinct sexual dimorphism: small, slightly oval spirals with 1.25 turns in females, 13–18 μm long and 9–12 μm wide; in males much larger, elongated loops, 28–38 μm long and 16–20 μm wide (Figs 5, 6, 12, 18, 28, 29).

**Figs. 27–31.**
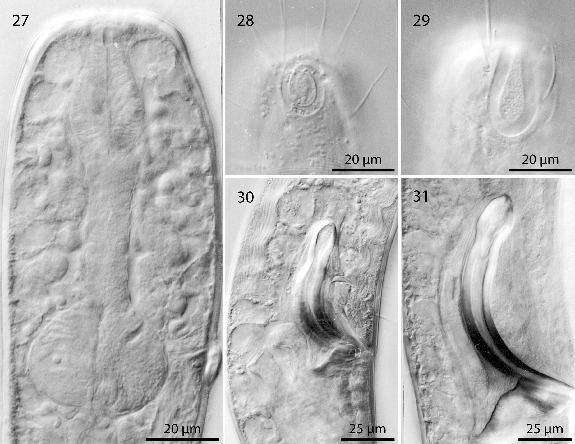
*Robbea hypermnestra* sp. nov. **27**. Pharyngeal region, optical section; **28**. Female, amphidial fovea; **29**. Male, amphidial fovea; **30**. Female, spiculum; **31**. Male, spiculum. LM Interference contrast.

Pharynx (Figs 4, 27) 90–117 μm long, with a minute tubular buccal cavity, 7–12 μm long and 4 μm in diameter, leading into a conspicuous pyriform muscular corpus, 34–40 μm long and 18–25 μm wide, which is clearly set off from the following 32–40 μm long isthmus. Posterior bulbus subspherical, 20–38 μm long, 30–45 μm wide, largely glandular, containing only weak muscles. No cardia.

Nerve ring 50–65 μm from anterior end; no secretory-excretory pore or ventral gland seen; 8 rows of glandular sense organs (two in each lateral and each median line) connect to the small somatic setae.

Males monorchic, testis on the left side of the intestine, beginning at about 35% of body length; sperm spherical, 10 μm in diameter; spicula (Figs 8, 10, 31) strong, arcuate, slightly cephalate proximally, with blunt tips (Fig. 24), 80–88 μm (chord) or 98–105 μm (arc) long; gubernaculum massive, corpus embracing spicules, 20–30 μm long, with strong dorsocaudal apophysis, 28–34 μm long).

Females didelphic, ovaries reflexed, long uteri leading to a strongly cuticularized vagina; vulva (Fig. 13) at 41–45% of body length. Large eggs (190 × 50 μm) are often present in one or both uteri, which also contain usually many sperm cells of the same shape and size as seen in males. All females are intersexes, having spicula and a gubernaculum in the anal region (Figs 7, 9, 30). Spicula and gubernaculum much smaller and of simpler construction than in males: length of the female spicula, 53–60 μm (chord) or 62–70 μm (arc); gubernaculum, 10–15 μm long with a similar sized apophysis. No traces of other parts of a male genital apparatus were found. A weakly cuticularized vulva primordium but no other female or male characteristic features could be seen in 7 of 11 large (probably 4th stage) juveniles.

The bacteria covering the cuticle of the worms (Fig. 14) are distributed over the whole body, leaving only the tip of the tail and the cephalic capsule uncovered. On the ventral side the bacteria rarely reach the basis of the cephalic capsule. The bacteria are rod shaped and are usually attached with their longitudinal axis perpendicular to the cuticle surface. In a few cases, however, bacteria were seen lying parallel to the cuticular annuli of the worm (Fig. 14). The thiotrophic bacterial ectosymbionts have been previously characterized (Bayer *et al.*, 2009) and belong to the MONTS clade of Gammaproteobacteria (Heindl *et al.,* 2011) that also contains the symbionts of the mouthless nematode genus Astomonema and of gutless oligochaetes. The GenBank accession number for their 16S rRNA gene is EU711428.

#### Diagnosis

Species with a distinct cephalic capsule with block-layer; 16–19 cup-shaped stalked ventral papillae in the post pharyngeal region; pharynx almost equally divided into corpus, isthmus and bulbus; all females are intersexes; spicula weakly cephalate, gubernaculum with dorsocaudal apophysis, pronounced sexual dimorphism of the amphidial fovea. Symbiotic bacteria rod-shaped.

***Robbea ruetzleri*** sp. nov.

http://zoobank.org/urn:lsid:zoobank.org:act:92A7E898-62D0-452D-A6A3-9A622CBD4599

#### Type material

Holotype (male), 3 paratypes (male), 3 paratypes (female), deposited at the US National Museum, Washington, D.C. (accession numbers USNM 1231560-1231566).

#### Measurements

See Table 2.

**Table 2.** Morphometric data for *Robbea ruetzleri* sp. nov. Ranges are given for the male and female paratypes. All measurements are in μm.

	Holotype	Paratypes (male) n = 3	Paratypes (female) n = 3
Length	2320	2280–3150	2750–3790
a	58.0	57.0–78.8	54.6–70.2
b	30.9	27.8–31.5	33.5–45.1
c	18.9	23.8–47.0	37.2–40.8
maximum width	40	40–45	50–60
pharynx length	75	72–90	72–78
tail length	72	70–88	69–75
nerve ring (% pharynx length)	53	55–60	55–58
corresponding body diameter (cbd)	38	35–40	38–42
bulbus length/width	12/17	15–17/19–20	12–18/18–20
bulbus cbd	35	34–39	33 –39
V	n.a.	n.a.	52–54
vulva cbd	n.a.	n.a.	50–60
anal (cloacal) body diameter	40	40–45	32–40
tail length:anal diameter	1.8	1.8–2.0	1.7–2.2
spiculae length arc/chord	75/60	72–85/60–66	n.a.
gubernaculum length	35	36–39	n.a.
amphid width	10	10–12	10–12
cephalic setae number/length	4/23	4/21–27	4/22–27
subcephalic setae 1 number/length	8/26	8/25–26	8/23–27
subcephalic setae 2 number/length	8/23	8/20–22	8/18–22
Sucker-like papillae, number	15	15–17	n.a.
Position of first/last papilla	72/315	80–105/305–415	n.a.

n.a. = not applicable.

#### Additional material

Several specimens in the authors’ collection and those used for SEM.

The sequence of the 18S rRNA gene is available from GenBank and has the accession number KJ414465.

#### Type locality

West side of Twin Cayes, Belize; shallow fine sand among *Rhizophora mangle* stilt roots and *Thalassia testudinum* beds (Fig. 1).

#### Distribution

Rare, in fine to medium subtidal sand samples (Fig. 1).

#### Etymology

Named in honour of Klaus Ruetzler, CCRE programme director, friend and generous host on Carrie Bow Cay.

### Description

**Figs. 32–36.**
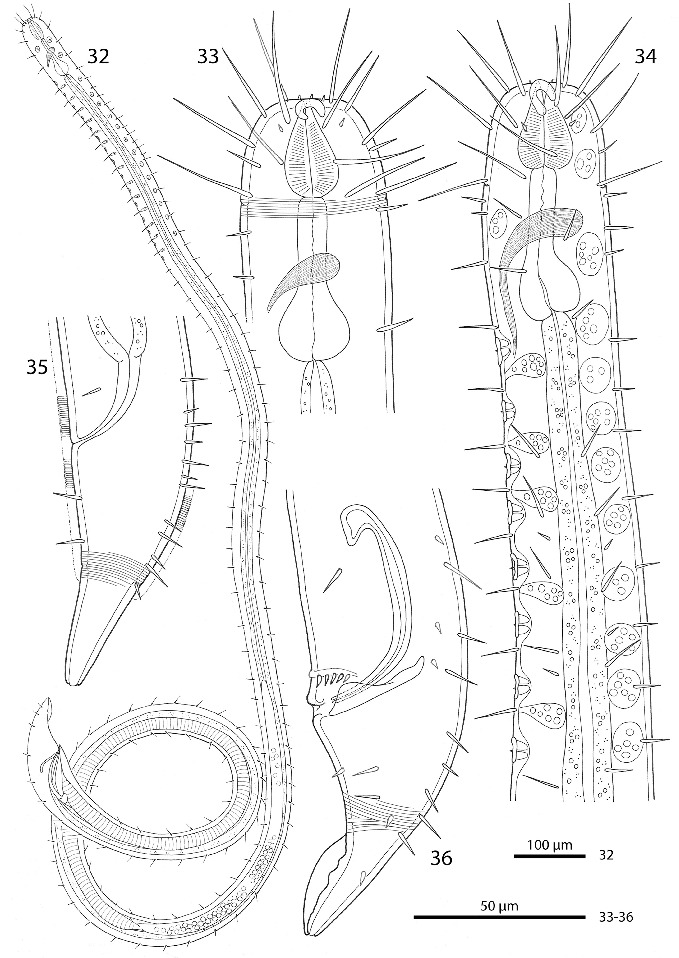
*Robbea ruetzleri* sp. nov. **32**. Male, whole; **33**. Female, anterior body region; **34**. Male, anterior body region; **35**. Female, tail; **36**. Male, tail and spicular apparatus.

Body slender, cylindrical (Fig. 32), head diameter at level of cephalic setae 20–25 μm, diameter at level of posterior margin of amphidial fovea 25–30 μm at end of pharynx 33–39 μm, maximum body diameter 40–60 μm, anal diameter 32–45 μm. Tail conical, 60–88 μm long (Figs 35, 36).

**Figs. 37–44.**
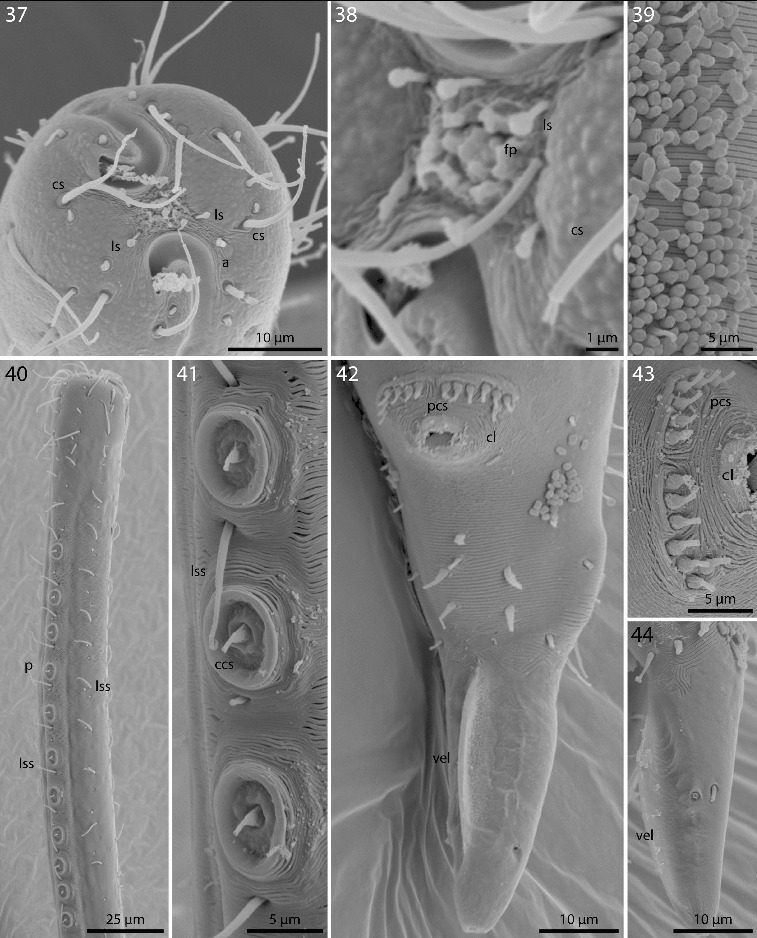
*Robbea ruetzleri* sp. nov. **37**. Male, head in face view; **38**. Mouth opening with fingerlike papillae and circle of outer labial sensillae; **39**. Annulation and bacterial coat in midbody region; **40**. Male, anterior body region with row of sucker-shaped papillae; **41**. Male, detail of papillae; **42**. Male, tail, cloaca and precloacal setae; **43**. Precloacal setae; **44**. Male, Tip of tail with velum. SEM.

Cuticle finely transversely striated except for the first 32–44 μm of the head and the last 30–42 μm of the tail, annules 0.5–0.65 μm wide (15–20 annuli/10 μm) (Figs 39, 42); head with a circle of 6 finger-like inner labial papillae surrounding the mouth opening (Fig. 38); 6 short outer labial sensillae, 2.5–4 μm long, at the margin of the membranous buccal field; 4 cephalic setae, flanking the anterior margin of the amphidial fovea, 21–27 μm long (Figs 33, 34, 37); a first circle of 8 subcephalic setae at the level of the amphidial fovea, a second circle approximately in the middle, a third circle at the posterior margin of the cephalic capsule; subcephalic setae, 18–27 μm long, 8 rows of somatic setae along the whole length of the body. Somatic setae of males in the region of the row of ventral papillae stout, 12–17 μm long, the following body setae thinner and shorter (8–13 μm). There is a transverse row of precloacal setae a short distance anterior to the cloacal opening (Figs 36, 42, 43). Somatic setae of females in cervical region 12–15 μm long, the following body setae 17–20 μm long. Males with a 250–320 μm long row of 15–17 conspicuous mid-ventral cuticularized papillae (Figs 34, 40), first papilla situated a short distance posterior to the end of the pharynx. Papillae with short annulated stalks, bearing central conical setae (Fig. 41). The non-striated part of the tail bears no terminal setae, in males there is a velum present (Figs 42, 44). Loop-shaped amphidial foveas (7–12 μm long, 10–12 μm wide), situated at the anterior end bordering the buccal field, with only slight sexual dimorphism.

**Figs. 45–47.**
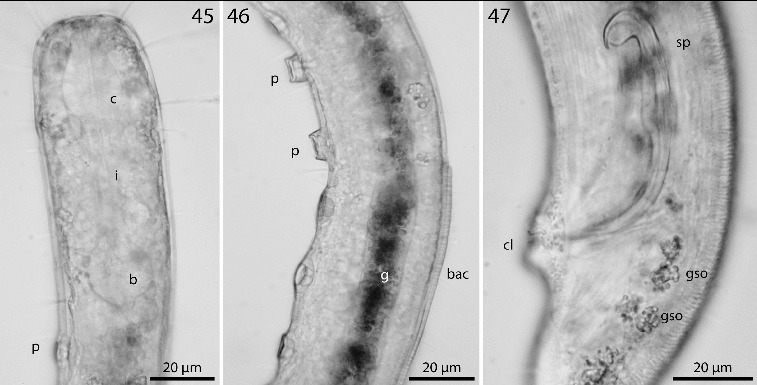
*Robbea ruetzleri* sp. nov. **45**. Pharyngeal region, optical section; **46**. Male, sucker-shaped papillae and begin of bacterial coat; **47**. Male, spiculum. LM of live animals.

Pharynx 72–90 μm long; minute tubular buccal cavity, 10–15 μm long and 2–4 μm in diameter, leading into a conspicuous pyriform muscular corpus, 22–30 μm long and 16–18 μm wide, clearly set off from the following 24–41 μm long isthmus. Spherical bulb, 12–18 μm long and 17–20 μm wide, mainly glandular and containing only weak muscles. No cardia (Figs 33, 34, 45).

Nerve ring 42–60 μm from anterior end; no secretory-excretory pore or ventral gland seen; there are 8 rows of glandular sense organs (two in each lateral and each median line) connecting to the small somatic setae.

Males monorchic, testis on the left side of the intestine, beginning at about 35% of body length; spicula strong, arcuate, distinctly cephalate proximally, 60–66 μm (chord) or 72–85 μm (arc) long, without velum; gubernaculum simple, embracing the distal part of the spicula laterally, with dorsally directed apophysis (35–37 μm long) (Figs 36, 47). Females didelphic, ovaries reflexed, long uteri leading to the vagina; vulva at 52–54 of body length.

A dense monolayer of rod-shaped symbiotic bacteria (Fig. 39) covers almost the whole body, beginning at the level of the pharyngeal terminal bulb or at the level of the posterior region of the cup-shaped cervical papillae (Fig. 46) and terminating with the cuticle striation at the tail tip or already at the level of the cloacal opening. Rows or plaques of smaller cocci stretch forward up to the anterior bulbus of the pharynx, but not up to the non-striated part of the head.

#### Diagnosis

Species with indistinct cephalic capsule; 14–17 cup-shaped stalked ventral papillae in the post pharyngeal region; pharynx almost equally divided into corpus, isthmus and bulbus; spicula strongly cephalate, gubernaculums without apophysis; amphidial fovea in both sexes open loop-shaped. Symbiotic bacteria rod shaped.

***Robbea agricola*** sp. nov.

http://zoobank.org/urn:lsid:zoobank.org:act:97995BAA-7914-4578-A4DE-64ED0DEF761B

#### Type material

Holotype (male), 3 paratypes (male), 3 paratypes (female), deposited at the US National Museum, Washington, D.C. (accession numbers USNM 1231567-1231573).

#### Additional material

Several specimens in the authors’ collection and those used for SEM.

#### Measurements

See Table 3.

**Table 3.** Morphometric data for *Robbea agricola* sp. n. Ranges are given for the male and female paratypes. All measurements are in μm.

	Holotype	Paratypes (male) n = 3	Paratypes (female) n = 3
Length	1880	1780–2550	2280–2550
a	47.0	40.0–54.3	57.0–68.2
b	19.8	19.6–28.7	28.0–37.4
c	22.1	18.8–21.3	27.3–29.6
maximum width	40	33–38	35–42
pharynx length	95	60–75	80–90
tail length	85	78–105	70–85
nerve ring (% pharynx length)	57	53–60	53–62
corresponding body diameter (cbd)	30	28–35	30–32
bulbus length/width	15/20	12–15/17–18	12–15/18–20
bulbus cbd	30	25–32	28–33
V	n.a.	n.a.	52–55
vulva cbd	n.a.	n.a.	35–42
anal (cloacal) body diameter	30	30–35	24–30
tail length:anal diameter	2.8	2.6–3.3	2.3–3.5
spiculae length arc/chord	55/40	45–58/35–45	n.a.
gubernaculum length/apophysis length	15/15	10–12/10–13	n.a.
amphid width	16	12–14	8–12
cephalic setae number/length	4/21	4/20–21	4/21–22
subcephalic setae 1 number/length	8/20	8/16 –20	8/20–26
subcephalic setae 2 number/length	8/16–18	8/13–16	8/18–22
Sucker-like papillae, number	9	9	n.a.
Position of first/last papilla	90/260	70–98/225–268	n.a.

n.a. = not applicable.

The sequence of the 18S rRNA gene is available from GenBank and has the accession number KJ414464.

#### Type locality

West side of Twin Cayes, Belize; shallow fine sand among *Rhizophora mangle* stilt roots and *Thalassia testudinum* beds (Fig. 1).

#### Distribution

Regularly in fine sand samples at several locations in the vicinity of the CBC laboratory (Fig. 1).

#### Etymology

Agricola (lat.) = farmer (German ‘Bauer’), named after Monika Bright (then Monika Bauer) who provided the first specimens.

### Description

**Figs. 48–54.**
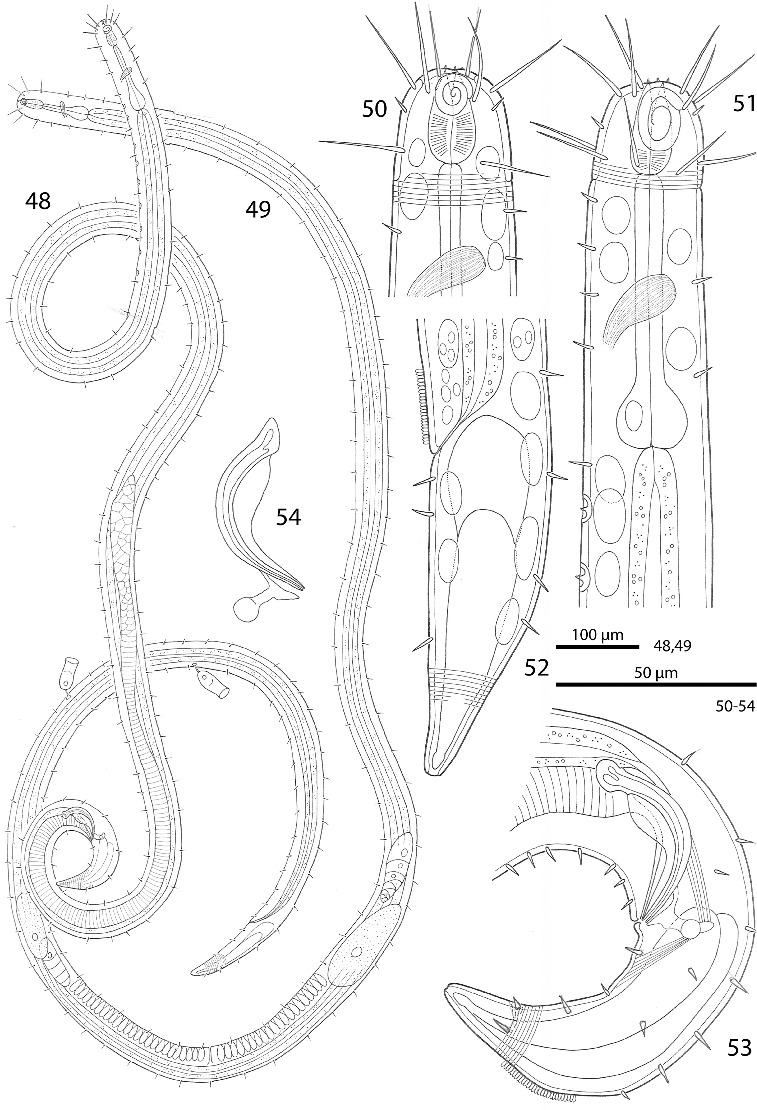
*Robbea agricola* sp. nov. **48**. Male, total; **49**. Female, total; **50**. Female, head region; **51**. Male, anterior body region; **52**. Female, tail; **53**. Male, tail; **54**. Spicular apparatus.

Body slender, cylindrical (Figs 48, 49), tapering only slightly towards anterior end, head diameter at level of cephalic setae 18–25 μm, diameter at level of posterior margin of amphidial fovea 20–29 μm, at end of pharynx 25–33 μm, maximum body diameter 33–42 μm, anal diameter 24–35 μm. Tail conical, 70–105 μm long; non-striated tail tip 17–19 μm long in males and 23–30 μm in females (Figs 52, 53, 62).

**Figs. 55–62.**
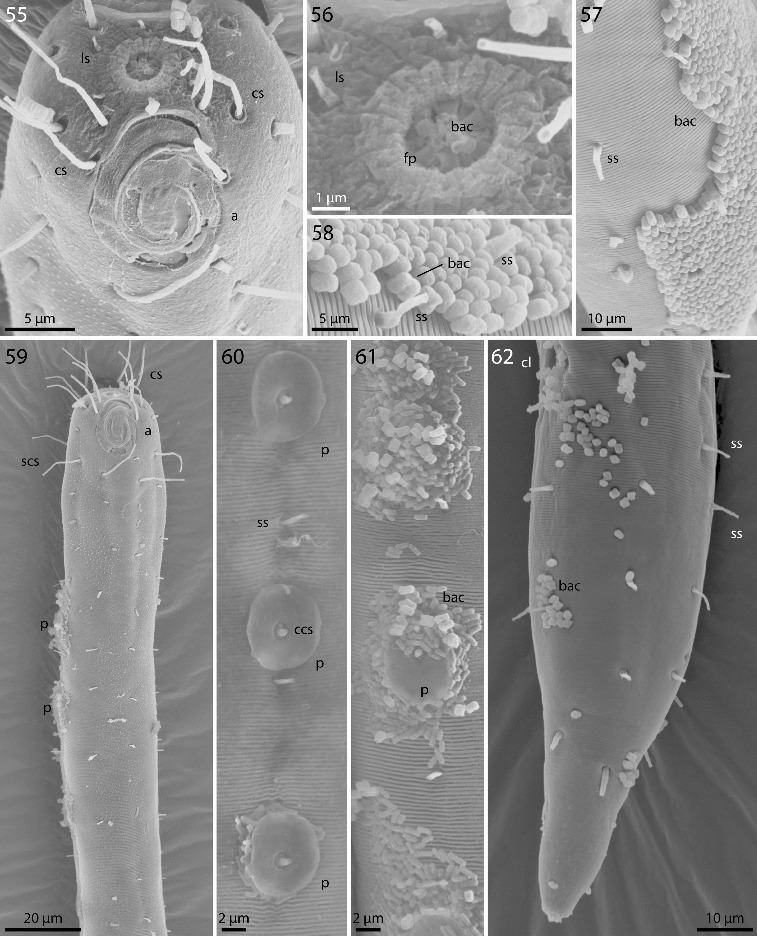
*Robbea agricola* sp. nov. **55**. Male, head in face view; **56**. Mouth opening with fingerlike papillae; **57**. Annulation and bacterial coat in midbody region; **58**. Coat of corn-kernel shaped bacteria; **59**. Male, anterior body region with the first part of the row of sucker-shaped papillae; **60**. Papillae; **61**. Papillae surrounded by bacterial growth; **62**. Female, tail. SEM.

Cuticle finely transversely striated except 24–28 μm of the anterior part of the head (cephalic capsule) and the tail tip, annules 0.6 to 0.7 μm wide (14–17 annuli/10 μm) (Fig. 57). Mouth opening surrounded by 6 finger-like inner labial papillae in lateral, subventral and subdorsal position (Fig. 56) followed by a circle of 6 short labial sensillae, 3–4 μm long, surrounding the membranous buccal field; a circle of 4 cephalic setae flanking the anterior margin of the amphidial fovea, 20–22 μm long; closely followed by a circle of 8 subcephalic setae, 16–26 μm long, a second circle near the end of the cephalic capsule (Figs 50, 51, 55); 8 rows of somatic setae along the whole length of the body, 5–8 μm long; a pair of 6–8 μm long setae at the begin of the non-striated part of the tail. No special precloacal setae. Mid-ventral line of males with a 150–170 μm long row of 8–9 conspicuous cup-shaped papillae (diameter 7 μm) positioned on short stalks. Papillae with central setae (1.5 μm long) (Figs 51, 59, 60). The first of these papillae is situated at a distance of 70–110 μm from the anterior end at the level of the pharyngeal terminal bulb. Amphidial foveas spiral with 2.5 turns, slight sexual dimorphism: in females oval, 12–13 μm long and 8–12 μm wide, situated directly at the anterior end bordering the buccal field; in males larger, somewhat elongated, 15–16 μm long, 12–14 μm wide, 1–4 μm from the anterior end (Figs 50, 51, 64).

**Figs. 63–65.**
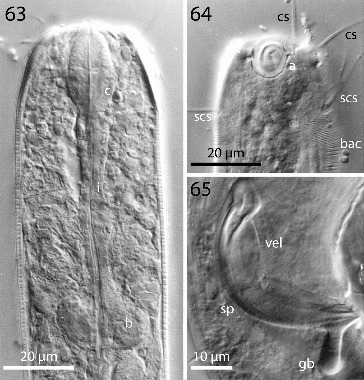
*Robbea agricola* sp. nov. 63. Pharyngeal region, optical section; **64**. Male, amphidial fovea; **65**. Male, spiculum. LM Interference contrast.

**Figs. 66–69.**
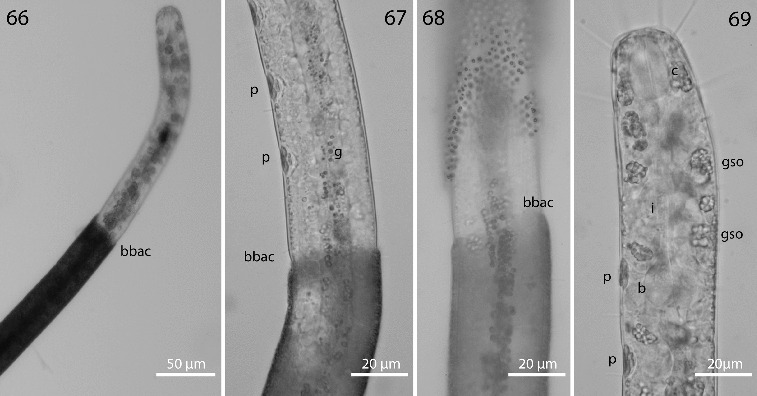
*Robbea agricola* sp. nov. **66**. Anterior body region and beginning of bacterial coat; **67**. Beginning of bacterial coat, detail; **68**. Large cocci on anterior body region; **69**. Glandular sensory organs. LM of live animals.

**Fig. 70.**
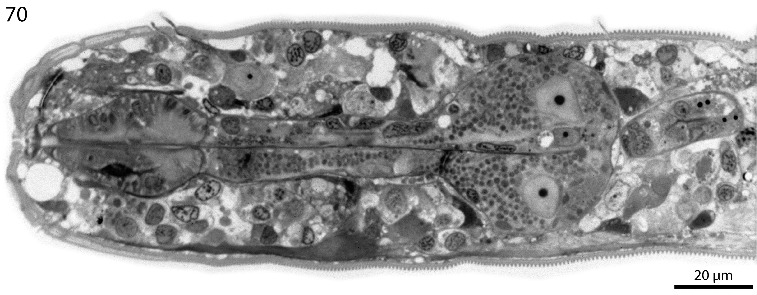
*Robbea hypermnestra* sp. nov. Semi-thin longitudinal section through pharynx showing separation of corpus from isthmus and glandular bulbus.

**Fig. 71.**
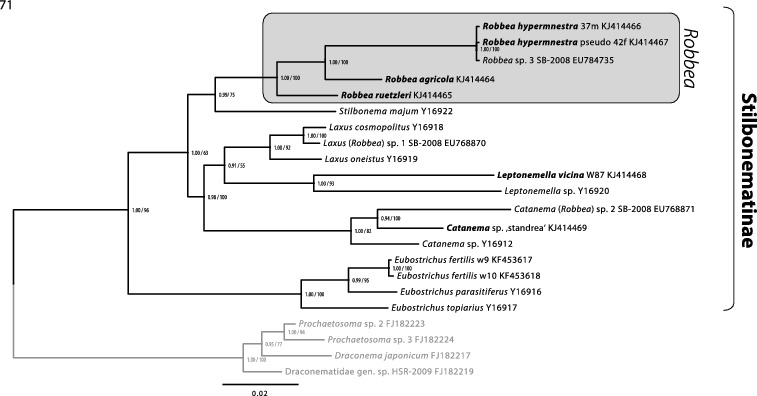
Phylogenetic relationships of the genera of the Stilbonematinae based on the 18S rRNA gene. The tree shown was calculated using maximum likelihood (RAxML) and node support is given as aLRT as well as Bayesian posterior probabilities. The scale bar indicates 0.02 nucleotide substitutions per site.

A minute tubular buccal cavity, 7–10 μm long and 2–3 μm in diameter, leading into a tripartite, 60–95 μm long pharynx consisting of an anterior pyriform muscular corpus (19–30 μm long, 12–18 μm wide), the following isthmus (30–50 μm long) and the terminal bulb (12–18 μm long, 17–20 μm wide). Terminal bulb largely glandular, containing only weak muscles. No cardia (Figs 50, 51, 63, 69).

Nerve ring 44–67 μm from anterior end; no secretory-excretory pore or ventral gland seen; 8 rows of glandular sense organs (two in each lateral and each median line) connecting to the small somatic setae.

Males monorchic, testis on the left side of the intestine, beginning at about 35% of body length; spicula strong, arcuate, slightly cephalate proximally, 35–45 μm (chord) or 55–58 μm (arc) long, with a velum; gubernaculum with a strong dorso-caudal directed apophysis (13–15μm long) ending in a spherical swelling (Figs 53, 54, 65).

Females didelphic, ovaries reflexed, long uteri leading to the vagina, ventral gso enlarged in the region of the uteri; vulva at 52–55% of body length.

Epigrowth of symbiotic bacteria starting at a defined line at a distance from the anterior end (Figs 66, 67) showing a sexual dimorphism: in males the bacterial coat begins at 220–340 μm (2.7–3.5 pharynx length), in females at 155–195 μm (2–2.3 pharynx length). A monolayer of corn-kernel shaped bacteria (1.5 × 0.8 μm) (Figs 57, 58) covers the remaining body except the non-striated tip of the tail. At the start of the coat the body diameter abruptly becomes smaller to accommodate the thickness of the bacterial layer without increasing the consortium's diameter (Fig. 67). In some specimens patches of bacteria occur around the cup-shaped papillae; here rods lie parallel to the cuticle surface (Fig. 61). Occasionally larger coccoid bacteria are found on the normally symbiont-free anterior body part (Fig. 68). The role of these bacteria is unknown.

Suctorians are frequently attached to the cuticle in the posterior body region (Fig. 49).

#### Diagnosis

Species with indistinct cephalic capsule; 8–9 cup-shaped ventral papillae in post-pharyngeal region; isthmus occupies more than 50% of pharynx length; spicula cephalate with velum, gubernaculums with dorso-caudal apophysis ending in a spherical swelling; amphidial fovea spiral in both sexes, larger in males. Symbiotic bacteria corn-kernel shaped, bacterial coat starting at a defined line 2 to 3.5 pharynx lengths from the anterior end.

## Discussion

### Systematics

Gerlach (1956) erected the genus *Robbea* for a male animal collected on the coast of Brazil, which he named *Robbea caelestis*. A second species was subsequently described from the Maldive Islands by the same author, *Robbea tenax* Gerlach (1963). The material consisted of one female and three males, the latter showing conspicuous cervical papillae. Both *Robbea* species were later placed into the genus *Catanema* Cobb, 1920 by Platt & Zhang (1982). Recently Tchesunov (2013) proposed diagnoses for both *Robbea* and *Catanema* in which the major distinguishing character is the shape of the amphidial fovea, which is distinct in *Robbea* but is reduced to a small apical opening in *Catanema*. Other characters such as the degree to which the pharyngeal corpus is set off from the isthmus, ‘clearly’ in *Robbea*, ‘distinctly’ in *Catanema* (see Fig. 70 for the situation in *Robbea hypermnestra*), and the presence (*Robbea*) or absence (*Catanema*) of a cephalic capsule are less clear and not all descriptions published so far contain explicit statements regarding these characters. According to Tchesunov's criteria, the following species belong to the genus *Robbea*: *R*. *caelestis* Gerlach 1956, *R. tenax* Gerlach 1963, *R. gallica* Vitiello 1974, R. (*Catanema*) *porosum* Hopper & Cefalu 1973, *R*. (*Catanema*) *macintyrei* Platt & Zhang 1982, *R*. (*Catanema*) *smo* Platt & Zhang 1982, furthermore the animal that Hopper & Cefalu (1973) described as *R. tenax* from Florida and the three new species described herein. *R. gerlachi* Boucher 1975 has only been described from a female, but according to the shape of its amphidial fovea it should be placed into the genus *Robbea.* One additional species of *Robbea* has been suggested by Tchesunov (2013), but due to the lack of male specimens the species has not been described in detail yet.

In the genus *Catanema* only two formally described species remain, the type species *C. exile* Cobb 1920 and *C. dambayensis* Tchesunov 2013. *Catanema cobbi* Inglis 1967 has been placed into the genus *Laxus* by Ott *et al*. (1995), *Catanema gerlachi sensu* Hopper & Cefalu 1973 most probably belongs to *Laxus cosmopolitus* (Ott *et al.*, 1995).

To consolidate the taxonomic descriptions of the three newly described *Robbea* species on a molecular level we used phylogenetic 18S rRNA analyses. The tree shows a clear separation between the clade containing the three new *Robbea* species and the other Stilbonematinae (Fig. 71). We also provide additional 18S rRNA gene sequences for the genera *Catanema* and *Leptonemella* that only had a single 18S rRNA gene deposited prior to this study. The morphological characters of the three yet undescribed *Catanema* species included in the phylogenetic analyses conform to the diagnosis given by Tchesunov (2013). Our data confirm that both *Robbea* and *Catanema*, as well as all other genera of Stilbonematinae with available sequence data are represented by statistically supported genus level clades in 18S rRNA gene based phylogenetic analyses. This high resolution of the 18S rRNA gene finally allows to assign or to re-evaluate the correct taxonomic affiliation at the genus level for new or already deposited sequences.

For the moment this ends the confusion around these two genera to which we have added by assigning two stilbonematine species to the genus *Robbea* (namely *Robbea* sp. 1 and 2) in Bayer *et al*. (2009). We have reinvestigated the nematode material used in that paper where possible. In the case of *Robbea* sp. 1 from Calvi (Corse), there obviously had been a mix-up during the sample sorting, and two different nematode species were present in the sample analysed: a *Laxus* sp. that is morphologically indistinguishable from *Laxus cosmopolitus* described from the Adriatic (Ott *et al*., 1995) and a yet undescribed *Catanema* species. Judging from the phylogenetic position of the deposited 18S rRNA gene, a specimen of the former species was probably sequenced. *Robbea* sp. 2 from the Cayman Islands is a member of the *Catanema* clade (Fig. 71). *Robbea* sp. 3 is morphologically and phylogenetically identical to the newly sequenced *Robbea hypermnestra* sp. nov. specimens (Fig. 71). The 18S sequences for *Robbea hypermnestra* sensu Kampfer 1998 (*nomen nudum*) that were formerly deposited under Y19621 were identified as chimeric but the last 600 bp are identical to the correct *Robbea hypermnestra* sp. nov. sequences from this study as well as to the *Robbea* sp. 3 sequence published by Bayer *et al*. (2009). The sequences in the Kampfer *et al*. (1998) paper were generated from pools of up to 50 nematodes that likely were contaminated by other nematode species. Thus, the chimeric nature of the published sequences could likely be attributed to different priming bias in the forward and reverse primers used. In contrast, all sequences generated in the present work come from single nematode specimens. This has been made possible with high-yield DNA extraction methods based on GeneReleaser (Bioventures) (Schizas *et al.*, 1997) or the Blood and Tissue kit (Qiagen) combined with highly sensitive and efficient polymerases such as the Phusion® DNA polymerase (Finnzymes). Successful PCR based sequencing of multiple genes from the DNA of a single meiofauna individual has been performed without amplification using specimens as small as 500 μm long microturbellarians (Gruber-Vodicka *et al.*, 2011). We thus emphasize that, wherever possible, single individuals should be used for PCR-based gene assays.

While the diagnoses given by Tchesunov (2013) currently hold true for the genera *Robbea* and *Catanema*, they are based on characters such as the reduction of the amphidial foveas. However, these characters are also present in some species of other genera, such as *Stilbonema* and *Leptonemella* (e.g. in the type species *L. cincta* COBB 1920). Findings of new species may make emendation of diagnoses that were based on morphology alone necessary. Our results clearly indicate the necessity to provide molecular data to confirm the morphological identification and that larger taxon sampling is an important factor to be able to validate sequencing results and enable for example barcoding approaches.

### Intersexes

Cases of intersexuality have been described from various terrestrial, parasitic or marine nematodes (e.g. Zhuo *et al.*, 2009; Moura *et al.*, 2014). In the latter, intersexes are most commonly reported as females with a functional reproductive system and rudimentary male sexual characters (e.g. Gourbault & Vincx, 1990; Riemann *et al.*, 2003; Zhuo *et al.*, 2009; Miljutina *et al.*, 2013; Moura *et al.*, 2014), just as observed in our newly described *R. hypermnestra*. However, only few intersex individuals are usually found within nematode populations (Gourbault & Vincx, 1990). As driving factor for intersexuality in nematodes, unfavourable environmental conditions (Davide & Triantaphyllou, 1968), hybridization between closely related species (Steiner, 1923; Krall, 1972) or genetic or chromosomal disorder (Roy & Gupta, 1975; Jairajpuri *et al.*, 1977) have been hypothesized. Considering the clean and stable environment around Carrie Bow Cay and the fact that all females of *R. hypermnestra* are intersexes, environmental sex determination appears unlikely. We cannot exclude hybridization events with closely related species in the past. It is difficult to assign any specific function to the spicula observed in *R. hypermnestra* intersexes and the *R. hypermnestra* chromosome set has not been determined, thus our hypotheses will remain rather speculative. The spicula could be remnants from a previously hermaphroditic lifestyle, but since no hermaphroditic lifestyle is known from stilbonematine nematodes, the interpretations that *R. hypermnestra* females are in a transition to a hermaphroditic stage, is more likely. In fact, individuals of the well-studied terrestrial nematodes *C. elegans* and *C. briggsae* are either male or hermaphrodites where the hermaphrodites are descendants from male or female ancestors and mutations in two independent pathways were sufficient for *C. elegans* to develop self-fertile hermaphrodites (Baldi *et al.*, 2009). This indicates a high plasticity and flexibility in the expression of sexual phenotypes in nematodes in general. Hermaphroditism could be favourable at low effective population densities to ensure reproduction in the absence of a mating partner (Pires-DaSilva, 2007). To test whether this is the case, the distribution patterns and gender ratios of the *R. hypermnestra* populations need to be monitored systematically.

### Ecological notes

The shallow subtidal sands around Carrie Bow Cay harbour a diverse interstitial meiofauna. Among these are representatives of several taxa known to harbour bacterial symbionts. These include the ciliate genus *Kentrophoros*, the mouthless nematode genus *Astomonema* (authors’ unpublished observation), several species of the mouthless catenulid flatworm genus *Paracatenula* (Dirks *et al.*, 2011; Gruber-Vodicka *et al.*, 2011) and the gutless marine oligochaete genera *Inanidrilus* and *Olavius* (Erséus, 1990). So far, stilbonematid nematode species of several genera have been described from shallow water habitats around Carrie Bow Cay. These include *Laxus oneistus*, *Stilbonema majum*, *Adelphus rolandi*, *Eubostrichus dianae*, *E.parasitiferus* and *E. fertilis* (Ott *et al.*, 1995, 2014; Ott, 1997; Kampfer *et al.*, 1998; Polz *et al.*, 1999). We add to this diversity with our description of three new *Robbea* species that live in vicinity of Carrie Bow Cay (Fig. 1). Available physiological and molecular evidence shows that all (*Paracatenula*, Stilbonematinae) or the majority of bacterial symbionts (oligochaetes) are sulphur-oxidizing chemoautotrophs (SOBs) (Dubilier *et al.*, 2006; Bayer *et al.*, 2009; Gruber-Vodicka *et al.*, 2011). Associations of SOBs with motile hosts appear to be beneficial when oxygen-containing surface layers are spatially separated from sulphidic deeper layers and vertical migration by the host can alternately supply the symbiotic bacteria with sulphide and oxygen (Giere *et al.*, 1991; Ott *et al.*, 1991).

Shallow sandbars in the immediate back reef area of Carrie Bow Cay consist of coarse to medium, unsorted carbonate sand of local origin that has been deposited under sheltered conditions and has a spacious interstitium with reduced, sulphidic conditions in layers several centimetres below the sediment surface (Ott & Novak, 1989). Here large species of Stilbonematinae (*Laxus oneistus*, *Stilbonema majum*) and also *R. hypermnestra* are found in high densities (Ott & Novak, 1989). In contrast, a diverse assemblage of smaller Stilbonematinae species such as *E. dianae*, *E. fertilis*, *R. agricola* and *R. ruetzleri* inhabit the fine sands in the vicinity of lagunal mangrove islands and *Thalassia testudinum* seagrass beds (Ott *et al.*, 2014). Additionally to the described species, several undescribed species and possibly genera of stilbonematine nematodes can be found in the shallow water habitats in the vicinity of Carrie Bow Cay (authors’ pers. observation).

## List of abbreviations in figures

a: amphidial fovea
b: pharyngeal bulbus
bac: bacteria
c: pharyngeal corpus
ccs: central conical setae
cg: corpus gelatum
cgo: caudal gland opening
cl: cloaca
cs: cephalic seta
fa: amphidial fovea fovea
fp: finger-like papillae
g: gut
gso: glandular sensory organ
i: pharyngeal isthmus
ls: labial sensillae
lss: long somatic setae
na: non-striated tail tip
p: sucker shaped papillae
pcs: precloacal setae
ss: somatic setae
scs: subcephalic seta
sp: spiculum
ss: somatic setae
ts: terminal seta
vel: velum
